# Autophagy caused by oxidative stress promotes TGF-β1-induced epithelial-to-mesenchymal transition in human peritoneal mesothelial cells

**DOI:** 10.1038/s41419-024-06753-z

**Published:** 2024-05-28

**Authors:** Se-Hyun Oh, Ju-Min Yook, Hee-Yeon Jung, Ji-Young Choi, Jang-Hee Cho, Sun-Hee Park, Chan-Duck Kim, Yong-Lim Kim, Jeong-Hoon Lim

**Affiliations:** 1 Division of Nephrology, Department of Internal Medicine, School of Medicine, Kyungpook National University, Kyungpook National University Hospital, Daegu, Republic of Korea; 2 Cell and Matrix Research Institute, Kyungpook National University, Daegu, Republic of Korea

**Keywords:** Chaperone-mediated autophagy, End-stage renal disease

## Abstract

Epithelial-to-mesenchymal transition (EMT) is one of the main causes of peritoneal fibrosis. However, the pathophysiological mechanisms of EMT, specifically its relationship with autophagy, are still unknown. This study aimed to evaluate the role of autophagy in transforming growth factor-beta 1 (TGF-β1)-induced EMT in human peritoneal mesothelial cells (HPMCs). Primary cultured HPMCs were treated with TGF-β1 (2 and 5 ng/mL) and changes in autophagy markers and the relationship between autophagy and EMT were evaluated. We also identified changes in EMT- and autophagy-related signaling pathways after autophagy and NADPH oxidase 4 (NOX4) inhibition. TGF-β1 increased the generation of NOX4 and reactive oxygen species (ROS) in HPMCs, resulting in mitochondrial damage. Treatment with GKT137831 (20 μM), a NOX1/4 inhibitor, reduced ROS in the mitochondria of HPMC cells and reduced TGF-β1-induced mitochondrial damage. Additionally, the indirect inhibition of autophagy by GKT137831 (20 μM) downregulated TGF-β1-induced EMT, whereas direct inhibition of autophagy using 3-methyladenine (3-MA) (2 mM) or *autophagy-related gene 5* (*ATG5*) gene silencing decreased the TGF-β1-induced EMT in HPMCs. The suppressor of mothers against decapentaplegic 2/3 (Smad2/3), autophagy-related phosphoinositide 3-kinase (PI3K) class III, and protein kinase B (Akt) pathways, and mitogen-activated protein kinase (MAPK) signaling pathways, such as extracellular signal-regulated kinase (ERK) and P38, were involved in TGF-β1-induced EMT. Autophagy and NOX4 inhibition suppressed the activation of these signaling pathways. Direct inhibition of autophagy and its indirect inhibition through the reduction of mitochondrial damage by upstream NOX4 inhibition reduced EMT in HPMCs. These results suggest that autophagy could serve as a therapeutic target for the prevention of peritoneal fibrosis in patients undergoing peritoneal dialysis.

## Introduction

Peritoneal dialysis (PD) utilizes the peritoneal membrane to exchange uremic toxins and water, effectively treating patients with end-stage kidney disease. However, long-term exposure to PD solutions with high concentrations of glucose and glucose degradation products causes functional and morphological changes to the peritoneum, which results in ultrafiltration failure [1, 2]. Mesothelial cells are the primary cells of the peritoneum, and their phenotypic transition is a crucial mechanism underlying peritoneal fibrosis [3, 4]. Following the initiation of PD, human peritoneal mesothelial cells (HPMCs) progressively lose their epithelial phenotype and acquire myofibroblast-like characteristics through epithelial-to-mesenchymal transition (EMT) [3]. Therefore, there has been considerable research interest in the pathophysiological mechanisms of EMT and its downregulation.

Reactive oxygen species (ROS) are generated in various intraperitoneal cells, including HPMCs, macrophages, and mast cells [5], and ROS generation is a well-known cause of peritoneal fibrosis in HPMCs [6]. High glucose concentrations increase the levels of transforming growth factor-beta 1 (TGF-β1), which upregulates cellular ROS through increased mitochondrial metabolism and NADPH oxidase (NOX) [7]. Various cell signaling pathways are involved in ROS-induced EMT; however, the precise mechanisms have yet to be elucidated [8].

Autophagy is a protein degradation system that regulates cellular homeostasis [9, 10]. Cytoplasmic components, including various proteins and organelles, are sequestered into double-membrane cytosolic vesicles called autophagosomes, which fuse with a lysosome for degradation and recycling [11, 12]. Autophagy regulates TGF-β1-induced fibrogenesis in primary human atrial myofibroblasts [13] and induces mesothelial cell transformation [14]. However, the role of autophagy and its relationship with oxidative stress in HPMCs remains uncertain. This study investigated the role of autophagy in the EMT of HPMCs caused by PD. This will help to identify the novel mechanisms underlying peritoneal dysfunction in PD patients and provide new therapeutic targets.

## Materials and Methods

### Primary culture of HPMCs

Omenta were obtained from patients who had undergone abdominal surgery. These patients provided informed consent to the use of their tissue in this study. HPMCs were isolated from the omenta using enzymatic disaggregation. For this, the cells were treated with trypsin-ethylenediaminetetraacetic acid 0.25% for 30 min in a water bath at 37 °C. The cultured HPMCs were seeded at 80% confluence and maintained in medium 199 (M199) supplemented with 20% fetal bovine serum (FBS) at 37 °C under 5% CO_2_ conditions. The primary cultured cells used were between passages 2 and 5.

### TGF-β1 treatment

The cells were plated at 70%–80% confluence and allowed to rest in 1% FBS-containing M199 for 24 h. The HPMCs were treated with 2 and 5 ng/mL of recombinant human TGF-β1 (R&D Systems, Inc., Minneapolis, MN, USA) for 48 h. They were then pretreated with the autophagy inhibitor, 2 mM 3-methyladenine (3-MA) (Selleckchem, Houston, TX, USA), or with 20 µM of the selective NOX1-NOX4 dual inhibitor, GKT137831 (Selleckchem), for 2 h at 37 °C.

### Cell viability assay

To study cell viability in the presence of 3-MA or GKT137831, cultured HPMCs were plated on 96-well plates and incubated with 1% FBS-containing M199 medium for 24 h. Subsequently, the cells were treated with 3-MA (1–10 mM) or GKT137831 (1–50 μM). Cell viability was analyzed using Cell Counting Kit-8 (CCK-8) (Dojindo Laboratories, Kumamoto, Japan) according to the manufacturer’s instructions. Absorbance was measured at 450 nm with a microplate reader (SPARK 10 M, Tecan, Durham, NC, USA). The results were expressed as percentages of the control value.

### Autophagy inhibition

To directly inhibit autophagy, HPMCs were pretreated with 3-MA and small interfering RNA (siRNA) targeting *ATG5*. HPMCs were pretreated with 3-MA (2 mM) for 2 h at 37 °C and then stimulated with TGF-β1 for 48 h. Human siRNA ATG5 and nontargeting (negative control) siRNA were purchased from Dharmacon (Chicago, IL, USA). The four target sequences in human ATG5 are 5′-GGCAUUAUCCAAUUGGUUU-3′, 5′- GCAGAACCAUACUAUUUGC-3′, 5 ′-UGACAGAUUUGACCAGUUU-3ʹ, and 5′- ACAAAGAUGUGCUUCGAGA-3′. Transfection was performed using Lipofectamine RNAiMax (Invitrogen, Paisley, Renfrewshire, UK) according to the manufacturer’s instructions. HPMCs (5.0 × 10^4^) were seeded 1 d prior to transfection and cultured to reach 40%–50% confluence by the following day. RNAi duplexes for *ATG5* were mixed with lipofectamine to form a transfection complex, which was added to the plated cells. All siRNAs were used at a concentration of 50 nM. After 6 h of transfection, the cells were incubated in M199 medium containing 1% FBS and treated with TGF-β1 (2 and 5 ng/mL) for an additional 48 h. A real-time polymerase chain reaction (PCR) was performed to confirm the effects of the siRNA transfection.

### NOX4 inhibition

For the inhibition of NOX4, a NOX1/4 dual inhibitor, GKT137831, was used. HPMCs were preincubated with 20 µM GKT137831 for 2 h before stimulation with TGF-β1 (2 and 5 ng/mL) to prevent ROS formation and then incubated with TGF-β1 for a further 48 h.

### Intracellular ROS measurement

The concentrations of intracellular ROS in the HPMCs were measured using a 2′,7′-dichlorofluorescin diacetate (DCF-DA)-Cellular ROS Assay Kit (Abcam, Cambridge, MA, USA), according to the manufacturer’s instructions. Briefly, cells were plated in 96-well black plates (1.5 × 10^4^ cells/well) and treated with TGF-β1 (2 and 5 ng/mL). After incubation for 24 h, 25 μM DCF-DA was added to each well and incubated for 45 min at 37 °C in dark conditions. Their fluorescence was measured using a microplate reader (SPARK 10 M, Tecan) at excitation and emission wavelengths of 485 and 535 nm, respectively. The fluorescence signal was expressed as a percentage of the control.

### Hydrogen peroxide assay

Extracellular H_2_O_2_ was measured using an Amplex Red Hydrogen Peroxide Assay Kit (Thermo Fisher Scientific, USA) according to the manufacturer’s instructions. Briefly, HPMCs were seeded in a 96-well black plate and treated with TGF-β1 (2 and 5 ng/mL). The H_2_O_2_ released from the treated HPMCs reacted with the Amplex Red reagent, in which horseradish peroxidase produced the red fluorescent oxidation product, resorufin. The resorufin fluorescence was measured using a fluorescence microplate reader (SPARK 10 M, Tecan) at excitation and emission wavelengths of 560 and 590 nm, respectively. The H_2_O_2_ concentrations were calculated using standard curves.

### Transmission electron microscopy

To compare the autophagy levels of HPMCs incubated for 48 h with or without TGF-β1 (2 and 5 ng/mL), we used transmission electron microscopy (TEM). After overnight fixation with 2.5% glutaraldehyde at 4 °C, the treated HPMCs were washed with phosphate-buffered saline (PBS) and post-fixed in 1% osmium (VIII) oxide (OsO_4_). The samples were then dehydrated in a series of graded ethanol solutions, embedded in Epon resin, and then cut into 60–80 nm-thick sections. These ultrathin sections were observed using a Hitachi HT7000 electron microscope (Tokyo, Japan).

### Autophagy flux assay

A Cyto-ID Autophagy Detection Kit (Enzo Life Sciences) was used to measure autophagy in HPMCs, according to the manufacturer’s protocol. Hoechst and Cyto-ID Green (Enzo Life Sciences) detection reagents (1:500 final dilution) were added directly to cells in an 8-well chamber slide. The cells were observed using a Zeiss Axio microscope. For plate reader measurements, the Hoechst and green detection reagent (1:1000 final dilution) solutions were added to cells grown in 96-well plates. Their fluorescence was measured using a microplate reader (SPARK 10 M, Tecan) at the excitation and emission wavelengths of 480 and 530 nm, respectively.

### RNA extraction and real-time quantitative RT-PCR

Total ribonucleic acid (RNA) was extracted from treated HPMCs using TRIzol reagent (Invitrogen, Waltham, MA, USA) according to the manufacturer’s instructions. The total RNA (1 µg) was reverse transcribed to complementary deoxyribonucleic acid (cDNA) using a PrimeScript cDNA Synthesis Kit (TaKaRa Shuzo Co. Ltd., Otsu, Japan). All primers for the quantitative reverse transcription PCR (qRT-PCR) (Suppl. Table 1) were designed using Primer Express v.1.5 (Applied Biosystems, Foster City, CA, USA) software. The qRT-PCR was performed, in duplicate, on an ABI PRISM 7500 Sequence Detection System (Applied Biosystems) using SYBR Green PCR Master Mix (Applied Biosystems). All samples were analyzed using the comparative Ct method (2^−ΔΔCt^) for the relative quantification of gene expression and normalization with respect to glyceraldehyde 3-phosphate dehydrogenase (GAPDH) expression.

### Immunofluorescence assay

HPMCs were plated on coverslips at appropriate densities of 70%–80%. Adhered cells were fixed with 4% paraformaldehyde for 10 min on ice and sealed with 10% bovine serum albumin (BSA) for 1 h. The cells were then incubated with microtubule-associated proteins 1 A/1B light chain 3B (LC3B) rabbit polyclonal antibody (0.5 µg/mL) (L10382, Invitrogen) for 1 h at room temperature (21 °C–23 °C). The autophagy-positive control was cells treated with 30 µM chloroquine for 16 h. The cells were incubated with Alexa Fluor 488-labeled goat anti-rabbit immunoglobulin G (H&L) (1:200) (ab150077, Abcam) for 30 min. To observe fibrosis, the cells were incubated overnight with α-SMA mouse monoclonal antibody (1:200) (A2547, Sigma-Aldrich, USA) at 4 °C. The second antibody was Alexa594-labeled goat anti-mouse immunoglobulin G (H&L) (1:200) (ab150116, Abcam) diluted in 1% BSA-PBS for 1 h. An anti-fluorescence quenching agent containing 4′,6-diamidino-2-phenylindole (DAPI) was applied to the coverslips. All microscopic images were recorded using either a confocal (Leica Stellaris 5, Leica Microsystems, Germany) or an automatic microscope.

### MitoSOX staining

MitoSOX Red selectively targets mitochondria and forms a stable fluorescent compound through superoxide oxidation. This was imaged using a green alter on a fluorescent microscope. For live-cell imaging, cells were stained with 5 mM MitoSOX™ Mitochondrial Superoxide Indicators (M36008, Invitrogen) for 10 min in an incubator, then washed twice with AM media. The live imaging was performed using an LSM 800 confocal microscope (Zeiss LSM 800 Laser Scanning Confocal Microscope SOP).

### Mitochondrial oxygen consumption rate measurement

Cells were seeded on a Seahorse XF 96-well plate overnight for the measurement of their oxygen consumption rate (OCR) and extracellular acidification rate (ECAR). Before measurement, the cells were washed and equilibrated for 1 h at 37 °C with XF base medium (102353-100; Agilent Technologies) supplemented with 1X GlutaMAX (35050; Gibco), 1 mM sodium pyruvate (S8636; Sigma-Aldrich), and 25 mM glucose (G7528; Sigma-Aldrich) (pH 7.4). A prehydrated sensor cartridge was loaded with the mitochondrial inhibitors to deliver a final concentration of 1 μM oligomycin (75351; Sigma-Aldrich), 1 μM carbonyl cyanide-p-trifluoromethoxy phenylhydrazone (FCCP) (C2920; Sigma-Aldrich), and 0.5 μM rotenone (R8875; Sigma-Aldrich) + 0.5 μM antimycin A (A8674; Sigma-Aldrich). This was placed on the XF 96-well plate, and the OCR was measured before and after the sequential injection of the mitochondrial inhibitors. For ECAR measurement, the cells were washed and the medium was replaced with glucose- or pyruvate-free XF base medium supplemented with 1 mM glutamine (G8540; Sigma-Aldrich) for 1 h before the assay. Subsequently, the cells were loaded with a sensor cartridge containing glucose, oligomycin (75351; Sigma-Aldrich), and 2-deoxyglucose (D6134; Sigma-Aldrich). These were injected at final concentrations of 10 mM, 2 μM, and 50 mM, respectively. The OCR and ECAR were measured using a Seahorse XFe 96 (Agilent Technologies) and analyzed using Wave 2.6.0 software after normalization to the total cell number.

### Western blot analysis

Cultured cells were lysed in Radio Immunoprecipitation Assay buffer (Pierce, Rockford, IL, USA) containing Xpert Protease Inhibitor Cocktail Solution (GenDEPOT, Baker, TX, USA). The protein concentrations in the lysates of the treated HPMCs were measured using Bradford’s method. The total protein (20 µg) was separated through 10% sodium dodecyl sulfate-polyacrylamide gel electrophoresis and transferred to a nitrocellulose membrane. The membrane was blocked with 10% skim milk for 1 h at room temperature (21 °C–23 ^o^C) and incubated overnight at 4 °C with the primary antibodies. The primary antibodies used were anti-Beclin 1 (Cell Signaling Technology, 3738; 1:1000), anti-p62 (Cell Signaling Technology, 5114; 1:1000), anti-LC3B (Cell Signaling Technology, 2775; 1:1000), anti-E-cadherin (Cell Signaling Technology, 3195; 1:1000), anti-fibronectin (Abcam, ab2413; 1:1000), anti-alpha-smooth muscle actin (α-SMA) (Sigma-Aldrich, A2547; 1:1000), anti-ATG5 (Cell Signaling Technology, 2630; 1:1000), anti-phospho-Smad2 (Millipore, 04-953; 1:1000), anti-Smad2 (Invitrogen, 51–1300; 1:1000), anti-phospho-Akt (Cell Signaling Technology, 4060; 1:1000), anti-phospho-Smad3 (Cell Signaling Technology, 9520; 1:1000), anti-Smad3 (Cell Signaling Technology, 9523; 1:1000), anti-Akt (Cell Signaling Technology, 2920; 1:1000), anti-phospho-PI3 kinase (PI3K) class III (Cell Signaling Technology, 13857; 1:1000), anti-PI3K class III (Cell Signaling Technology, 4263; 1:1000), anti-phospho-p38 (Abcam, ab4822; 1:1000), anti-p38 (Abcam, ab31828; 1:1000), anti-phospho-ERK (Abcam, ab4819; 1:1000), anti-ERK (Abcam, ab17942; 1:1000), anti-phospho-c-Jun N-terminal kinases (JNK) (Abcam, ab4821; 1:1000), anti-JNK (Abcam, Ab179461; 1:1000), and anti-GAPDH (Cell Signaling Technology, 2118; 1:2000). After washing, the membrane was incubated with horseradish peroxidase-conjugated secondary antibodies (P0447 and P0448) (Dako, Glostup, Denmark) for 1 h. Finally, enhanced chemiluminescence (ECL) reagents (Amersham Bioscience, Piscataway, NJ, USA) were used to allow visualization of the membrane on an ImageQuant™ LAS 4000 system (GE Healthcare Life Sciences, Tokyo, Japan). The protein band densities were quantified using Scion Image v.4.0 (Scion, Frederick, MD, USA) software.

### Statistical analysis

Mann-Whitney *U*-tests were used to compare the mean values between two groups as the sample size was small. Statistical analyses were performed using SPSS v. 22.0 (IBM Corp., Armonk, NY, USA). *P*-values < 0.05 were considered statistically significant.

### Ethics statement

The study protocol was reviewed and approved by the Institutional Review Board of the Kyungpook National University Hospital (KNUH-2016-04-002-001). The study was conducted in accordance with the principles of the 2013 revision of the Declaration of Helsinki. Written informed consent to specimen use and study publication was obtained from all patients who provided omentum specimens.

## Results

### TGF-β1 induces EMT and ROS generation in HPMCs

**Fig. 1:**
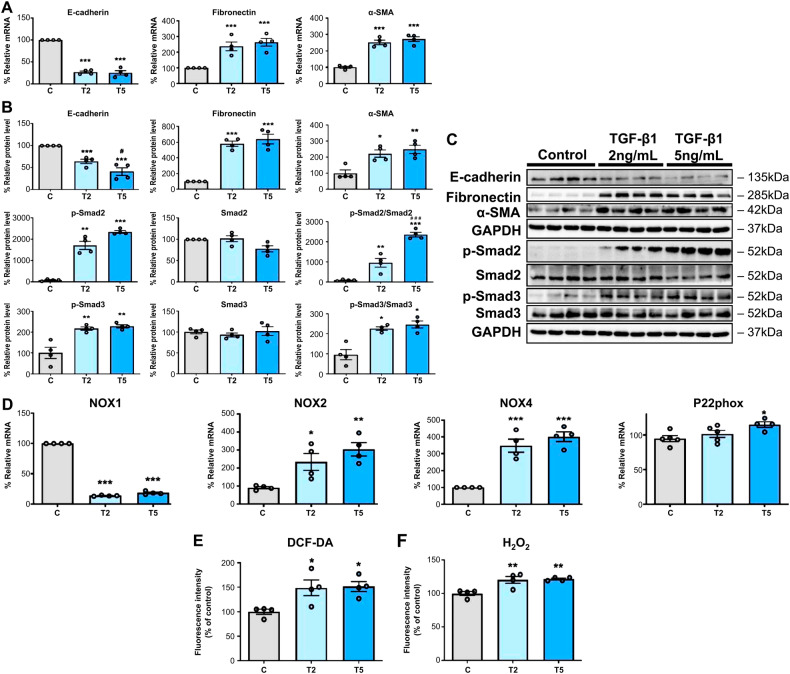
Increased epithelial-to-mesenchymal transition (EMT) via activation of the Smad2/3 signaling pathways and NOX2/4-induced ROS generation in HPMCs treated with TGF-β1. **A** TGF-β1 treatment (2 [T2] and 5 [T5] ng/mL) increased mRNA expression of the profibrotic mesenchymal markers (E-cadherin, fibronectin, and α-SMA) in HPMCs. **B**, **C** TGF-β1 treatment (2 and 5 ng/mL) increased protein levels of the profibrotic mesenchymal markers and activated the phosphorylation of Smad2/3 signaling in HPMCs. **D** TGF-β1 treatment (2 and 5 ng/mL) increased the mRNA expression of *NOX2/4* and *P22phox* in HPMCs after 48 h. **E** TGF-β1 induced ROS generation, which was measured using DCF-DA 1 h after TGF-β1 treatment (2 and 5 ng/mL). **F** TGF-β1 induced H_2_O_2_ generation 24 h after treatment (2 and 5 ng/mL). The data are presented as mean ± standard error. *n* = 4 per group. ^*^P < 0.05 vs. control (**C**); ^**^P < 0.01 vs. control; and ^***^P < 0.001 vs. control.

Treatments with 2 and 5 ng/mL TGF-β1 decreased the messenger RNA (mRNA) expression of the epithelial cell marker, E-cadherin, and increased that of mesenchymal markers such as fibronectin and α-SMA (Fig. 1A). Consequently, the protein expression of E-cadherin decreased and that of fibronectin and α-SMA increased after treatment with TGF-β1. This indicated that TGF-β1 had induced EMT in the HPMCs (Fig. 1B, C). Similarly, the protein expression levels of phosphorylated Smad2 (p-Smad2), p-Smad2/Smad2, phosphorylated Smad3 (p-Smad3), and p-Smad3/Smad3 were substantially increased (Fig. 1B, C). These results indicated that treatment with TGF-β1 induces fibrosis in HPMCs by upregulating the Smad2/3 signaling pathways.

The changes in oxidative stress markers and the NOX homologs, NOX1, 2, and 4, after treatment with TGF-β1 were also evaluated. An increase in NOX expression in TGF-β1-exposed HPMCs is one of the earliest indications of oxidative stress injury and EMT [15]. Treatment with TGF-β1 upregulated *NOX2* and *NOX4* and downregulated *NOX1* mRNA expression (Fig. 1D). Specifically, *NOX4* mRNA was significantly higher after 2 and 5 ng/mL TGF-β1 treatments than in the control group. P22phox is a NOX membrane partner and is essential for NOX-induced ROS generation [16]. The mRNA expression of *P22phox* was upregulated after treatment with 5 ng/mL TGF-β1 (Fig. 1D). Treatment with 2 and 5 ng/mL TGF-β1 increased ROS generation in HPMCs, as determined using intracellular ROS (DCF-DA) and H_2_O_2_ assays (Fig. 1E, F).

### TGF-β1 activates autophagy in HPMCs

**Fig. 2:**
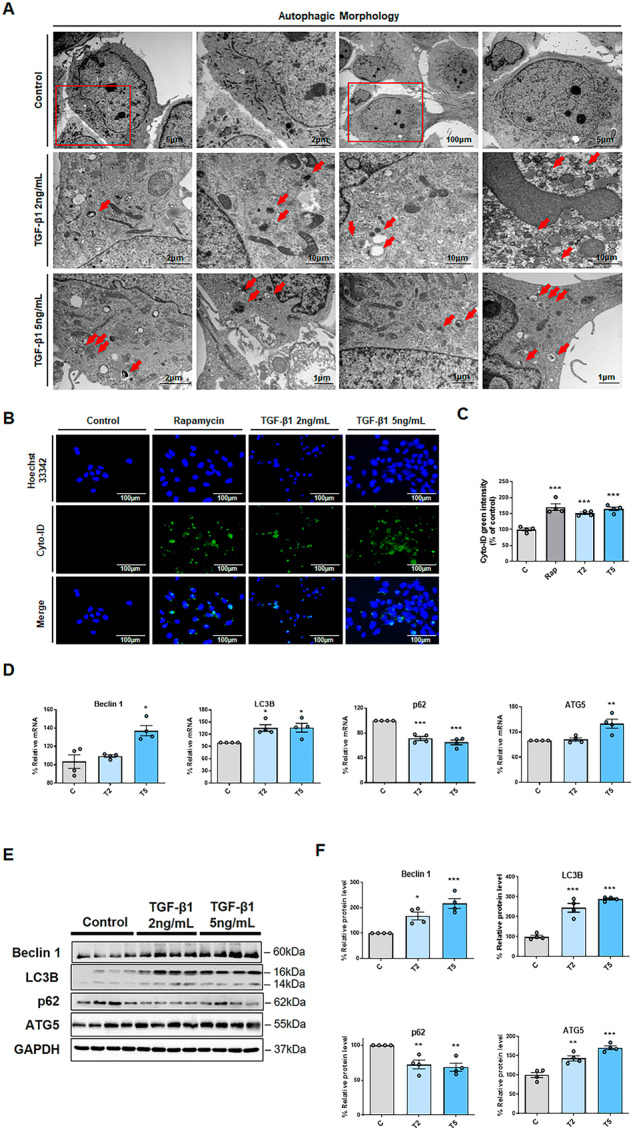
TGF-β1 induced autophagy activation in HPMCs, which was confirmed using transmission electron microscopy (TEM), an autophagy flux assay, and western blotting analysis. **A** Representative TEM images of the autophagic morphology. Autophagosomes were frequently observed after TGF-β1 treatment (2 and 5 ng/mL) in HPMCs. The red boxed portion is shown at high magnification on the right. The red arrow indicates the autophagosomes. **B** TGF-β1-induced autophagy was evaluated by staining using a Cyto-ID Autophagy Detection Kit. Rapamycin (0.5 μM; Rap) was used as a positive control. The stained cells were observed and photographed under fluorescence microscopy (blue, nucleus/ Hoechst 33342; green, autophagosomes/ Cyto-ID). **C** The intensity of the Cyto-ID green was quantified using a plate reader. **D** TGF-β1 (2 and 5 ng/mL) increased the mRNA expression of *Beclin 1*, *LC3B*, and *ATG5*, and decreased the mRNA expression of p62. (**E**, **F**) TGF-β1 (2 and 5 ng/mL) increased the protein levels of Beclin 1, LC3B, and ATG5, and decreased the protein levels of p62. The results were calculated as values relative to the control. Data are presented as mean ± standard error (SE). *n* = 4 per group. ^*^P < 0.05 vs. control; ^**^P < 0.01 vs. control; and ^***^P < 0.001 vs. control.

TEM images confirmed an increase in the formation of autophagosomes in HPMCs after treatment with TGF-β1 (Fig. 2A). TGF-β1-activated autophagy was also confirmed using autophagy flux assays (Fig. 2B). Autophagy flux increased after treatment with 2 and 5 ng/mL TGF-β1 to a similar degree to that observed in the positive control treated with 0.5 μM rapamycin (Fig. 2C).

Changes in individual markers of autophagy were identified. The mRNA expression and protein levels of the autophagy initiation marker Beclin 1 and autophagosome markers LC3B and ATG5 were significantly increased after treatment with TGF-β1 (Fig. 2D–F). Conversely, the mRNA expression and protein levels of p62, an autophagy regulator, decreased after TGF-β1 treatment. This indicated that TGF-β1 increased the degradation of p62. These results consistently indicate that autophagy activation increased after treatment with TGF-β1.

### Autophagy inhibition downregulates TGF-β1-induced EMT in HPMCs

**Fig. 3:**
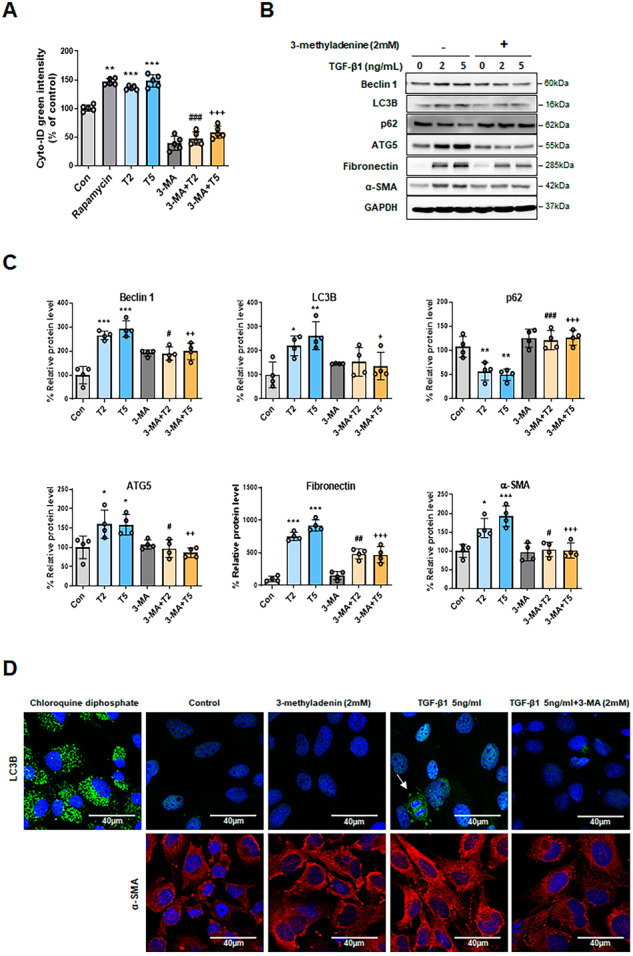
The effects of 3-methyladenine (3-MA) on TGF-β1-induced autophagy activation and epithelial-to-mesenchymal transition (EMT) in HPMCs. **A** TGF-β1 (2 and 5 ng/mL) induced autophagy activation but co-treatment with 3-MA (2 mM; 3-MA + T2 and 3-MA + T5) decreased autophagy activation. This was confirmed using a Cyto-ID Autophagy Detection Kit. **B**, **C** 3-MA treatment (2 mM) suppressed TGF-β1 (2 and 5 ng/mL) and induced autophagy activation. This was confirmed by western blotting analysis, which revealed decreases in Beclin 1, LC3B, and ATG5, and an increase in p62 levels. Protein levels of the mesenchymal markers fibronectin and α-SMA were decreased by 3-MA in TGF-β1-treated HPMCs. **D** Representative immunofluorescence images showing LC3 staining of TGF-β1-induced autophagy activation and EMT in HPMCs. (**D**, upper phase) Representative immunofluorescence images showing LC3B staining of TGF-β1-induced autophagy activation. Positive control cells were treated with 30 µM chloroquine for 16 h. Arrows indicate autophagic flux. (**D**, lower phase) Representative immunofluorescence images showing α-SMA staining of TGF-β1-induced EMT. Arrows indicate the lamellipodia. Scale bar = 40 μm. The data are presented as mean ± standard error (SE). *n* = 4 per group. ^*^P < 0.05 vs. control; ^**^P < 0.01 vs. control; ^***^P < 0.001 vs. control; ^#^*P* < 0.05 vs. TGF-β1 2 ng/mL; ^##^*P* < 0.01 vs. TGF-β1 2 ng/mL; ^###^*P* < 0.001 vs. TGF-β1 2 ng/mL; ^+^*P* < 0.05 vs. TGF-β1 5 ng/mL; ^++^*P* < 0.01 vs. TGF-β1 5 ng/mL; and ^+++^*P* < 0.001 vs. TGF-β1 5 ng/mL.

To confirm whether autophagy was associated with EMT, we identified changes in TGF-β1-induced EMT after autophagy inhibition. When cells were pretreated with 2 mM of the autophagy inhibitor 3-MA, the activation of autophagy flux was significantly lower than after treatment with TGF-β1 alone (2 and 5 ng/mL) (Fig. 3A). Cotreatment with 3-MA (2 mM) and TGF-β1 (2 and 5 ng/mL) led to the downregulation of autophagy activation marker proteins, including Beclin 1, LC3B, and ATG5, and the upregulation of autophagy inactivation marker protein p62. This was confirmed by western blot analysis (Fig. 3B, C). The protein levels of fibrosis markers such as fibronectin and α-SMA were significantly lower in HPMCs cotreated with 3-MA and TGF-β1 than those treated with TGF-β1 alone. Immunofluorescence staining revealed that intracellular autophagy formation was reduced by treatment with 3-MA (Fig. 3D). Pretreatment with the autophagy inhibitor 3-MA (2 mM) resulted in substantially less autophagic flux activity than treatment with TGF-β1 alone (5 ng/mL) (Fig. 3D, upper phase). Additionally, fibrosis markers were increased by treatment with TGF-β1 alone (5 ng/mL), and decreased by autophagy inhibition (Fig. 3D). These results indicate that intracellular autophagy is part of the fibrosis induction mechanism.

We confirmed the effects of autophagy inhibition through *ATG5* gene silencing, which codes for an essential protein in phagophore membrane extensions in autophagic vesicles. After *ATG5* silencing in HPMCs, TGF-β1-induced protein levels of Beclin 1, LC3B, and ATG5 were downregulated, whereas those of p62 were upregulated, indicating the inactivation of autophagy (Suppl. Fig. S1). The TGF-β1-induced increase in mesenchymal marker protein levels, such as fibronectin and α-SMA, was reduced after the *ATG5* gene was silenced.

These results confirmed that autophagy inhibition prevents TGF-β1-induced EMT and fibrosis in HPMCs, although the autophagy inhibition mechanisms are different.

### NOX4 inhibition downregulates TGF-β1-induced autophagy and EMT in HPMCs

We evaluated the effects of GKT137831, a NOX 1/4 inhibitor, on the upregulation of ROS generation, autophagy, and EMT induced by TGF-β1 treatment. TGF-β1 (2 and 5 ng/mL) was found to increase ROS generation 15 min, 30 min, 1 h, and 2 h after treatment, whereas cotreatment with GKT137831 (20 μM) reduced ROS generation (Supplementary Fig. S2).

**Fig. 4:**
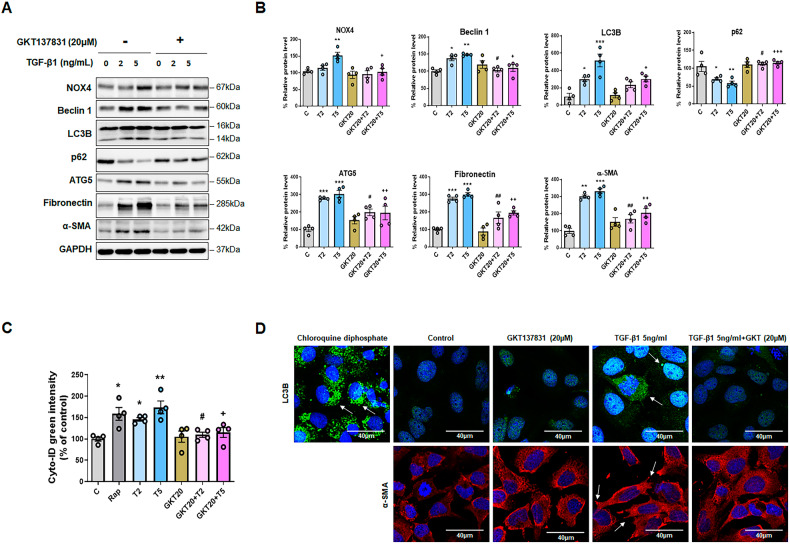
The effects of NOX4 inhibition on TGF-β1-induced autophagy activation and epithelial-to-mesenchymal transition (EMT) in HPMCs. **A** The expression of NOX4 and autophagy marker protein levels following GKT137831 (20 μM; GKT20) and TGF-β1cotreatment. **B** GKT137831 (20 μM) co-treatment resulted in lower NOX4 protein levels than TGF-β1 (5 ng/mL) treatment alone. Cotreatment with GKT137831 (20 μM) suppressed TGF-β1-induced autophagy activation. This was confirmed by western blotting analysis, which revealed decreased Beclin 1, LC3B, and ATG5, and increased p62 levels. The protein levels of the mesenchymal markers fibronectin and α-SMA were decreased by GKT137831 in TGF-β1-treated HPMCs. **C** TGF-β1 (2 and 5 ng/mL) induced autophagy activation and co-treatment with GKT137831 (20 μM; GKT20 + T2 and GKT20 + T5) decreased autophagy activation. This was confirmed using a Cyto-ID Autophagy Detection Kit. **D** Representative immunofluorescence images showing LC3B staining of TGF-β1-induced autophagy activation (upper phase). Positive control cells were treated with 30 µM chloroquine for 16 h. The arrows indicate autophagic flux. Representative immunofluorescence images showing α-SMA staining of TGF-β1-induced EMT (lower phase). The arrows indicate the lamellipodia. Scale bar = 40 μm. Data are presented as mean ± standard error (SE). *n* = 4 per group. ^*^P < 0.05 vs. control; ^**^P < 0.01 vs. control; ^***^P < 0.001 vs. control; ^#^*P* < 0.05 vs. TGF-β1 2 ng/mL; ^##^*P* < 0.01 vs. TGF-β1 2 ng/mL; ^+^*P* < 0.05 vs. TGF-β1 5 ng/mL; ^++^*P* < 0.01 vs. TGF-β1 5 ng/mL; and ^+++^*P* < 0.001 vs. TGF-β1 5 ng/mL.

NOX4 protein levels increased after treatment with TGF-β1 (5 ng/mL) but decreased with GKT137831 (20 μM) pretreatment (Fig. 4A, B). Protein levels of the autophagy markers Beclin 1, LC3B, and ATG5 were lower, and those of p62 were higher, after cotreatment with GKT137831 than after treatment with TGF-β1 alone, indicating the downregulation of autophagy (Fig. 4A, B). Furthermore, protein levels of fibronectin and α-SMA were significantly lower after cotreatment with GKT137831 and TGF-β1 than after treatment with TGF-β1 alone (2 and 5 ng/mL).

Cotreatment with GKT137831 (20 μM) and TGF-β1 decreased autophagy flux more than TGF-β1 treatment alone (2 and 5 ng/mL) (Fig. 4C). Intracellular autophagy formation (LC3B) and fibrosis (α-SMA) were observed via immunofluorescence staining. Cotreatment with GKT137831 (20 μM) and TGF-β1 also reduced autophagic flux activity (Fig. 4D, upper phase). Importantly, cotreatment with GKT137831 (20 μM) and 3-MA (2 mM) did not result in the evidence of fibrosis, such as lamellipodia, observed when TGF-β1 (5 ng/mL) was administered alone (Fig. 4D).

These results indicated that NOX4 suppression inactivates TGF-β1-activated autophagy and that autophagy is a critical mechanism in HPMC fibrosis caused by NOX4-derived ROS.

### NOX4 inhibitor and autophagy inhibition reduces mitochondrial damage in TGF-β1-induced HPMCs

**Fig. 5:**
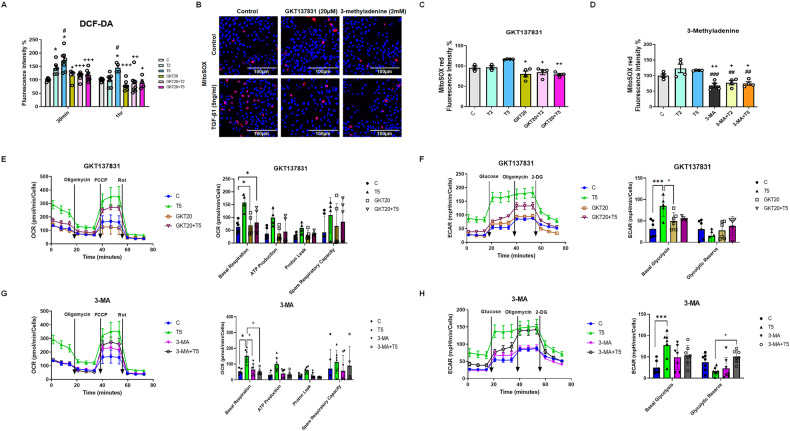
The reduction of oxidative phosphorylation and glycolysis by a NOX4 inhibitor in TGF-β1-induced HPMCs. **A** The determination of ROS production using DCF-DA (*n* = 4). **B** Mitochondrial superoxide production in live cells was measured using fluorescence microscopy with MitoSOX Red dye. Representative fluorescence images showing the localization of MitoSOX Red fluorescence. Scale bar = 40 μm. **C**, **D** The level of MitoSOX Red fluorescence per cell was quantified using ImageJ software. Image data from 51–60 cells per treatment condition were averaged (*n* = 3). **E**, **F** The measurement of mitochondrial oxygen consumption ratio (OCR) and extracellular acidification rates (ECAR) under NOX4 inhibition with GKT137831-treated HPMCs. The parametric indices of OCR and ECAR, mitochondrial respiration, and glycolysis are shown (*n* = 4–6). **G**, **H** The measurement of mitochondrial OCR and extracellular acidification rates (ECAR) under autophagy inhibition by 3-MA treatment. The parametric indices of OCR and ECAR, mitochondrial respiration, and glycolysis are shown. (*n* = 4–7). The data are presented as mean ± standard error (SE). ^*^P < 0.05 vs. control, ^***^P < 0.001 vs. control; ^#^*P* < 0.05 vs. TGF-β1 2 ng/mL; ^##^*P* < 0.01 vs. TGF-β1 2 ng/mL; ^###^*P* < 0.001 vs. TGF-β1 2 ng/mL; ^+^*P* < 0.05 vs. TGF-β1 5 ng/mL; ^++^*P* < 0.01 vs. TGF-β1 5 ng/mL; and ^+++^*P* < 0.001 vs. TGF-β1 5 ng/mL.

Based on preliminary experiments, we found TGF-β1 to significantly increase the generation of ROS (H_2_O_2_ and DCF-DA) after 30 mins and 1 h. Intracellular ROS levels were assessed using DCF-DA fluorescence. These levels showed a 170% increase when HPMCs were treated with 5 ng/mL of TGF-β1 (Fig. 5A). To determine the origin of the ROS, 20 μM GKT137831, a NOX4 inhibitor, was added. This significantly reduced ROS generation in the TGF-β1-treated cells (Fig. 5A). TGF-β1 was also found to increase MitoSOX expression. In addition to the mitochondrial respiratory chain, cellular oxidases, including NADPH oxidases such as NOX4 are also important sources of cellular ROS. After 48 h of TGF-β1 exposure, MitoSOX red fluorescence intensity was decreased by GKT137831 (20 μM) treatment (Fig. 5B, C). The production of TGF-β1-induced mitochondrial ROS was also reduced by the administration of 2 mM 3-MA (Fig. 5D). Treatment with 20 μM GKT137831 resulted in the same reduction in TGF-β1-induced mitochondrial ROS as that produced by the potent autophagy inhibitor, 3-MA.

We investigated cellular respiration in HPMCs by evaluating the OCR and ECAR. To determine the maximum oxidative capacity, we injected oligomycin into the culture wells followed by the unbinding agent FCCP. TGF-β1 (5 ng/mL) consistently raised the basal OCR after 48 h. The OCR reached its highest point in a dose-response pattern, and this increase was associated with the generation of adenosine triphosphate (ATP), an OCR sensitive to oligomycin, total oxidative capacity, and an OCR sensitive to FCCP. After 48 h exposure to TGF-β1 (5 ng/mL), the oxidative capacity of cells was higher than that of the control group (Fig. 5E, G). Treatment with GKT137831 (20 μM) reduced the TGF-β1-induced increase in oxidative capacity (Fig. 5E). This followed the same trend as the results obtained with the autophagy inhibitor 3-MA (2 mM) (Fig. 5G). GKT137831 (20 μM) reduced the abnormal respiration rate of mitochondria induced in control cells by TGF-β1 (5 ng/mL) and exhibited similar mitochondrial protection to that of direct autophagy inhibition with 3-MA (2 mM) (Fig. 5E, G). Additionally, using a Seahorse XF glycolysis stress test (Agilent Technologies), we found that TGF-β1-treated (5 ng/mL) HPMCs displayed a higher ECAR than control cells (Fig. 5F, H). A parameter analysis revealed significantly enhanced glycolysis and glycolytic capacity in the TGF-β1-induced (5 ng/mL) HPMCs. This suggests that TGF-β1-induced EMT progression causes more rapid glucose use via the glycolytic pathway in HPMCs. GKT137831 (20 μM) reduced the TGF-β1-stimulated glycolysis to a more normal level than 3-MA (2 mM) (Fig. 5F, H).

### Autophagy inhibition suppresses TGF-β1-induced activation of the Smad2/3, PI3K/Akt, and MAPK signaling pathways

We evaluated changes in the activation of the fibrosis-related Smad2/3, autophagy-related PI3K/Akt, and redox-sensitive MAPK pathways (ERK/P38/JNK) after autophagy inhibition to identify the signaling mechanisms underlying autophagy-mediated EMT. Autophagy was found to be inhibited by 3-MA and *ATG5* gene silencing.

**Fig. 6:**
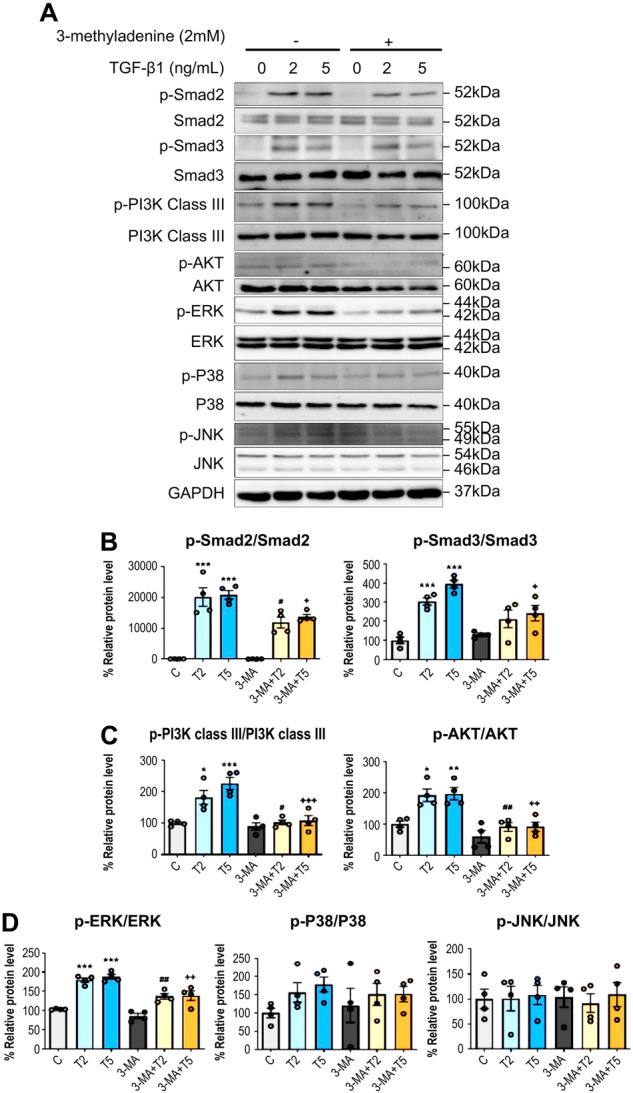
Autophagy inhibition by 3-MA treatment inactivated the Smad2/3, PI3K/Akt, and ERK pathways in HPMCs. 3-MA treatment (2 mM) decreased the TGF-β1 (2 and 5 ng/mL)-induced phosphorylation of Smad2/3 signaling for EMT (**A**, **B**), PI3K and Akt signaling for autophagy (**A**, **C**), and the ERK signaling of the MAPK pathway (**A**, **D**). The data are presented as mean ± standard error (SE). *n* = 4 per group. ^*^P < 0.05 vs. control; ^**^P < 0.01 vs. control; ^***^P < 0.001 vs. control; ^#^*P* < 0.05 vs. TGF-β1 2 ng/mL; ^##^*P* < 0.01 vs. TGF-β1 2 ng/mL; ^+^*P* < 0.05 vs. TGF-β1 5 ng/mL; ^++^*P* < 0.01 vs. TGF-β1 5 ng/mL; and ^+++^*P* < 0.001 vs. TGF-β1 5 ng/mL.

TGF-β1 treatment (2 and 5 ng/mL) resulted in increased phosphorylation of Smad2/3, PI3K class III, Akt, and ERK. Figure 6A–D illustrate the changes in signaling pathways after 3-MA treatment. 3-MA treatment (2 mM) significantly reduced the TGF-β1-induced phosphorylation of Smad2/3, PI3K class III, Akt, and ERK. Among the MAPK signaling pathways, the phosphorylation levels of P38 and c-Jun N-terminal kinase (JNK) did not change after treatment with TGF-β1, either with or without 3-MA.

Silencing of the *ATG5* gene revealed that TGF-β1 treatment (2 and 5 ng/mL) increased the phosphorylation of Smad2/3, PI3K class III, Akt, ERK, and P38 (Suppl. Fig. S3). As with 3-MA-induced autophagy inactivation, *ATG5* gene silencing significantly reduced the TGF-β1-induced phosphorylation of Smad2/3, PI3K class III, Akt, ERK, and P38. However, JNK phosphorylation in the MAPK signaling pathways was unchanged after both 3-MA treatment and autophagy inactivation by *ATG5* gene silencing.

### NOX4 inhibition suppresses TGF-β1-induced activation of the Smad2/3, PI3K/Akt, and MAPK signaling pathways

**Fig. 7:**
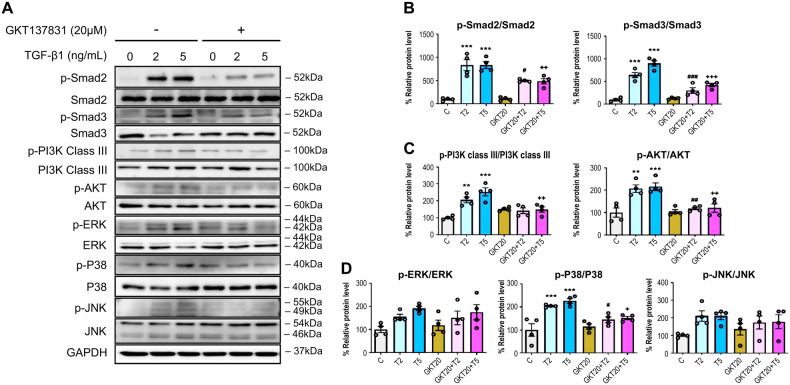
NOX4 inhibition with GKT137831 inactivated the Smad2/3, PI3K/Akt, and P38 pathways in HPMCs. GKT137831 (20 μM) decreased the TGF-β1 (2 and 5 ng/mL)-induced phosphorylation of Smad2/3 signaling for EMT (**A**, **B**), PI3K and Akt signaling for autophagy (**A**, **C**), and the P38 signaling of the MAPK pathway (**A**, **D**). The data are presented as mean ± standard error (SE). *n* = 4 per group. ^*^P < 0.05 vs. control; ^**^P < 0.01 vs. control; ^***^P < 0.001 vs. control; ^#^*P* < 0.05 vs. TGF-β1 2 ng/mL; ^##^*P* < 0.01 vs. TGF-β1 2 ng/mL; ^###^*P* < 0.001 vs. TGF-β1 2 ng/mL; ^+^*P* < 0.05 vs. TGF-β1 5 ng/mL; ^++^*P* < 0.01 vs. TGF-β1 5 ng/mL; ^+++^*P* < 0.001 vs. TGF-β1 5 ng/mL.

We identified the signaling mechanisms related to NOX4-mediated autophagy activation and EMT. Treatment with TGF-β1 (2 and 5 ng/mL) increased the phosphorylation of Smad2/3, PI3K class III, Akt, and JNK (Fig. 7). Specifically, it significantly increased the phosphorylation of P38, which is involved in mitochondrial dysfunction. Conversely, treatment with GKT137831 (20 μM) significantly reduced the TGF-β1-induced phosphorylation of Smad2/3, PI3K class III, Akt, and P38. Among the MAPK signaling pathways, ERK and JNK phosphorylation remained unchanged after TGF-β1 treatment, either with or without GKT137831.

These changes in the downregulation of the signaling pathways were similar to those after 3-MA treatment and *ATG5* gene silencing, both of which directly inhibit autophagy. These findings indicate that NOX4 activation triggers mitochondrial dysfunction and autophagy, which serve as the primary pathophysiological mechanisms behind EMT in HPMCs.

## Discussion

This study demonstrates the importance of autophagy in TGF-β1-induced EMT in HPMCs. TGF-β1 treatment activates autophagy through increased NOX4-derived ROS generation, resulting in mitochondrial damage. Both direct inhibition of the autophagy pathway and indirect suppression of autophagy through NOX4 inhibition reduced TGF-β1-induced EMT in HPMCs. These findings suggest autophagy inhibition as a potential therapeutic strategy for the prevention of peritoneal fibrosis and the preservation of peritoneal membrane function in patients undergoing PD.

EMT is a complex process involving multiple biochemical changes. These allow epithelial cells to acquire mesenchymal-like phenotypes characterized by increased migratory capacity and invasiveness [1, 17]. TGF-β1 is generated by high levels of glucose and glucose degradation products in the PD fluid and has been identified as a prominent contributor to peritoneal EMT among patients undergoing PD, which culminates in the loss of peritoneal membrane function [6]. Previous studies have shown that an increase in ROS via NOX activation by TGF-β1 is essential to the progression of peritoneal EMT [18, 19]. Nonetheless, the exact mechanism by which ROS triggers EMT has not previously been determined. Therefore, we elucidated the effect of autophagy activation on TGF-β1-induced EMT, particularly in the context of NOX4-derived ROS-induced EMT.

Autophagy is an intracellular degradation process that occurs in all mammalian cells and is essential to eliminate cellular waste components such as cytoplasm, organelles, and damaged proteins [20, 21]. The stages of autophagy are initiation, elongation, maturation, formation of the double-membrane autophagosome, fusion with lysosomes to form autolysosomes, and degradation [22, 23]. Autophagy can exert either protective or detrimental effects on cells. In pleural mesothelial cells, autophagy protects against EMT. A study has shown that impairment of autophagy by NOX4 increases EMT, and that NOX4 is induced by mycobacterial infection [19]. In the present study, NOX4 expression was induced by TGF-β1 treatment and autophagy activation resulted in EMT in HPMCs. Following TGF-β1 treatment, the levels of autophagy initiation markers (Beclin 1) and autophagosome markers (LC3B and ATG5) increased, indicating heightened autophagy activity. To establish the link between autophagy and EMT, we inhibited autophagy using 3-MA and *ATG5* gene silencing. The results demonstrated that the inhibition of autophagy decreases EMT, suggesting that autophagy is associated with and contributes to EMT. The differing results of our study and previous research on the role of autophagy in the EMT of mesothelial cells suggest that the role of autophagy may vary depending on the location and type of cell and the degree of cellular stimulation, even within the same mesothelial cell.

Interestingly, our findings suggest that activation of autophagy by TGF-β1 is indirectly induced by increased ROS within the mitochondria and mitochondrial damage. Two previous studies have reported that a high-glucose dialysis solution and the consequent production of TGF-β1 induces autophagy activation, promoting peritoneal fibrosis [2, 24]. However, these studies only identified the relationship between autophagy activation, fibrosis, and apoptosis. They did not reveal the relationships between autophagy and upstream pathways, such as NOX-ROS, mitochondrial damage, and changes in the related signaling pathways. Nor did they evaluate the effect of indirect autophagy inhibition through the inhibition of upstream pathways. Therefore, their interpretation of the inhibitory effects of autophagy was limited. Moreover, we confirmed the close relationship between NOX-ROS-induced mitochondrial damage and autophagy activation, confirming its effect on fibrosis by inhibiting the various pathways involved in each step.

NOX4 was first identified as a renal-specific NOX that is abundantly expressed in endothelial and vascular smooth muscle cells [25, 26]. NOX4 activation produces ROS, and NOX4-derived ROS plays a pivotal role in autophagy activation in various cells [27, 28]. The inhibition of NOX4 by GKT137831 decreased ROS generation and NOX4 expression. Subsequently, this reduced autophagy activation and the downregulation of EMT markers. Therefore, NOX4-derived ROS is vital to this process, connecting autophagy and EMT. We further identified a novel mechanism of EMT via mitochondrial damage and autophagy activation caused by NOX4-derived ROS generation. The PI3K class III signaling pathway is a key regulator of mitochondrial dysfunction [29] and mitochondrial apoptosis [30]. Treatment with GKT137831 reduced TGF-β1-activated ROS and mitochondrial dysfunction through PI3K class III phosphorylation. This demonstrates that NOX4 significantly promotes oxidative stress and mitochondrial dysfunction in TGF-β1-induced fibrosis.

**Fig. 8:**
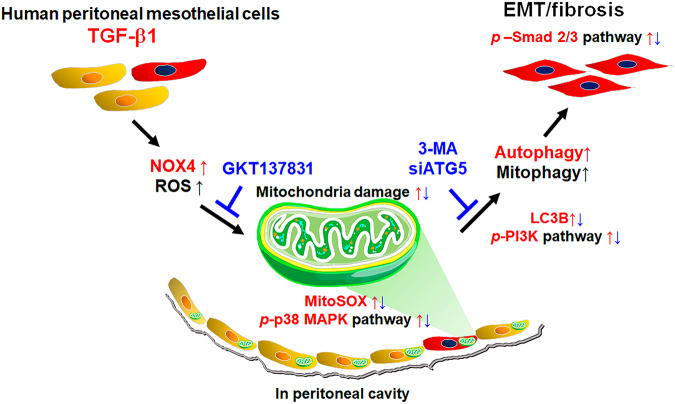
A summary of the results of the present study. In HPMCs, TGF-β1-induced NOX4 activation resulted in increased reactive oxygen species (ROS) generation, leading to mitochondrial damage. Increased ROS within the mitochondria activates the PI3K/Akt pathway, induces mitochondrial damage, and promotes the activation of autophagy. Autophagy activation promotes epithelial-to-mesenchymal transition (EMT) in HPMCs via the Smad2/3, PI3K/Akt, and ERK/P38 pathways. Both direct inhibition of autophagy by 3-MA or *ATG5* gene silencing, and indirect inhibition of autophagy through NOX4 inhibition by GKT137831, ameliorated the TGF-β1-induced EMT in HPMCs. Upregulation is shown in red and downregulation in blue.

Our study explores the effect of autophagy inhibition on various signaling pathways, including Smad2/3, PI3K/Akt, and MAPK. Autophagy inhibition reduced the phosphorylation of Smad2/3, PI3K class III, Akt, and ERK, suggesting that autophagy plays a role in the activation of signaling pathways that contribute to EMT. We also investigated the effect of NOX4 inhibition on the signaling pathways. GKT137831 reduced Smad2/3, PI3K class III, Akt, and P38 phosphorylation, further supporting the role of NOX4 in activating these pathways and leading to EMT. Figure 8 summarizes these results. TGF-β1 activated NOX4 expression, and NOX4 generated ROS in HPMCs. We confirmed that mitochondrial damage and autophagy activation are mechanisms by which ROS induces EMT. Various signaling pathways are involved in TGF-β1-induced NOX4-ROS-mitochondrial damage-autophagy-EMT in HPMCs.

This study had several limitations. First, only in vitro experiments were performed. Follow-up studies are required to confirm the association between autophagy and peritoneal fibrosis as well as the effect of autophagy inhibition. Second, we did not identify changes in the sub-pathways of the signaling pathways. Third, autophagy can have protective and damaging effects, and we were unable to compare the effects of gradual autophagy activation on fibrosis in detail. Therefore, further research is required.

In conclusion, TGF-β1-induced autophagy activation has a detrimental effect on the promotion of fibrosis in HPMCs. The upstream mechanism of autophagy activation is a TGF-β1-induced NOX4 increase, in which NOX4 increases the generation of intramitochondrial ROS, resulting in mitochondrial dysfunction. Mitochondrial damage triggers the activation of mitophagy and autophagy. The inhibition of autophagy, either by directly inhibiting autophagy or indirectly reducing mitochondrial damage through upstream NOX4 inhibition, reduces EMT in HPMCs. Elucidating the novel autophagy-related mechanisms of EMT will further our understanding of the mechanisms behind peritoneal fibrosis. We suggest that autophagy may be a potential therapeutic target for such fibrosis.

### Supplementary information

Supplementary Material

Original Data File

## Data Availability

The data that support the findings of this study are available in the methods and/or supplementary material of this article.
