# Supplementary material for: Autophagy caused by oxidative stress promotes TGF-β1-induced epithelial-to-mesenchymal transition in human peritoneal mesothelial cells
Source: Cell Death Dis. 2024 May 28;15(5):365. doi: 10.1038/s41419-024-06753-z (PMC11133371; doi:10.1038/s41419-024-06753-z)
Supplement: Supplementary file 1 — Supplementary Material [file 41419_2024_6753_MOESM1_ESM.docx]

**SUPPLEMENTARY MATERIAL**

**Supplementary Table 1.** Primer sequences used for quantitative RT-PCR

| Target | Primer | Sequences |
| --- | --- | --- |
| Human Beclin 1 | Forward  Reverse | 5′-GAG CTG GAA GAC GTG GAA AAG A-3′  5′-GCC TGG ACC TTC TCG AGA TTT-3′ |
| Human *LC3B* | Forward  Reverse | 5′-GGC GCT TAC AGC TCA ATG C -3′  5′-ATG CTG TGT CCG TTC ACC AA-3′ |
| Human p62 (*SQSTM1*) | Forward  Reverse | 5′-GAG CGG CTC TGG ACA CCA T-3′  5′-TGG GCA AAA GTG GTC ACA AC-3′ |
| Human *ATG5* | Forward  Reverse | 5′-TGA CAA AGA TGT GCT TCG AGA TG-3′  5′-ATA GTA TGG TTC TGC TTC CCT TTC A-3′ |
| Human E-cadherin | Forward  Reverse | 5′-GGC CTG AAG TGA CTC GAT ACG A-3′  5′-CAG CCG CTT TCA GAT TTT GAT C-3′ |
| Human Fibronectin | Forward  Reverse | 5′-CCA AGA AGG GCT CGT GTG A-3′  5′-GGC TGG AAC GGC ATC AAC-3′ |
| Human *NOX1* | Forward  Reverse | 5′-TGC CTA GAA GGG CTC CAA AC-3′  5′-ACA TTC AGC CCT AAC ACA AC-3′ |
| Human *NOX2* | Forward  Reverse | 5′-AGG GTC AAG AAC AGG CTA AGG A-3′  5′-TTC TCC ACC TCC AAC CCT CTT T-3′ |
| Human *NOX4* | Forward  Reverse | 5′-AAG CCA GTC ACC ATC ATT TCG-3′  5′-CTT TGA CCA TTC GGA TTT CCA-3′ |
| Human *P22phox* | Forward  Reverse | 5′-ACTTTGGTGCCTACTCCATTGTG-3′  5′-TGTCCCCAGCGCTCCAT-3′ |

**Supplementary Figure S1.**





**Figure S1.** Effect of *ATG5* gene silencing on TGF-β1-induced autophagy activation and EMT in HPMCs. (**A**, **B**) *ATG5* gene silencing suppressed the TGF-β1 (2 [siATG5+T2] and 5 [siATG5+T5] ng/mL)-induced autophagy activation, which was confirmed via Western blotting analysis with a decrease in the ATG5, Beclin 1, and LC3B levels and an increase in the p62 levels. Protein levels of the mesenchymal markers (fibronectin and α-SMA) were decreased by *ATG5* gene silencing in TGF-β1-treated HPMCs. The data are presented as the mean ± standard error (SE); n = 4 per group. ^*^*P* < 0.05 vs. control small interfering RNA (siCon); ^**^*P* < 0.01 vs. siCon; ^***^*P* < 0.001 vs. siCon; ^#^*P* < 0.05 vs. siCon+TGF-β1 2 ng/mL (siCon+T2); ^##^*P* < 0.01 vs. siCon+T2; ^###^*P* < 0.001 vs. siCon+T2; ^+^*P* < 0.05 vs. siCon+TGF-β1 5 ng/mL [siCon+T5]; ^++^*P* < 0.01 vs. siCon+T5; and ^+++^*P* < 0.001 vs. siCon+T5.

**Supplementary Figure S2.**


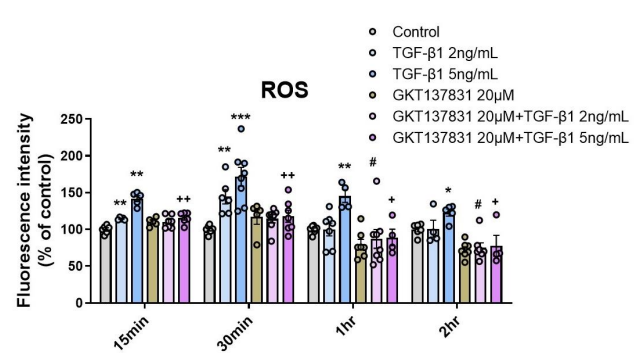


**Figure S2.** NOX4 inhibition ameliorates TGF-β1-induced ROS generation in HPMCs. TGF-β1 treatment (2 and 5 ng/mL) increased the generation of ROS at 15 min, 30 min, 1 h, and 2 h in the HPMCs. The GKT137831 co-treatment (20 μM) reduced the ROS assessed using DCF-DA. The data are presented as the mean ± standard error (SE). ^*^*P* < 0.05 vs. control; ^**^*P* < 0.01 vs. control; ^***^*P* < 0.001 vs. control; ^#^*P* < 0.05 vs. TGF-β1 2 ng/mL; ^+^*P* < 0.05 vs. TGF-β1 5 ng/mL; and ^++^*P* < 0.01 vs. TGF-β1 5 ng/mL.

**Supplementary Figure S3.**





**Figure S3.** Autophagy inhibition by *ATG5* gene silencing inactivates the Smad2/3, PI3K/AKT, and ERK pathways in HPMCs. *ATG5* gene silencing decreased the TGF-β1 (2 [siATG5+T2] and 5 [siATG5+T5] ng/mL)-induced phosphorylation of Smad2/3 signaling for EMT (**A**, **B**), PI3K and AKT signaling for autophagy (**A**, **C**), and ERK and P38 signaling of the MAPK pathway (**A**, **D**). The data are presented as the mean ± standard error (SE); n = 4 per group. ^*^*P* < 0.05 vs. control small interfering RNA (siCon); ^**^*P* < 0.01 vs. siCon; ^***^*P* < 0.001 vs. siCon; ^#^*P* < 0.05 vs. siCon+TGF-β1 2 ng/mL (siCon+T2); ^##^*P* < 0.01 vs. siCon+T2; ^+^*P* < 0.05 vs. siCon+TGF-β1 5 ng/mL (siCon+T5); ^++^*P* < 0.01 vs. siCon+T5; and ^+++^*P* < 0.001 vs. siCon+T5.
