# Supplementary material for: Autophagy caused by oxidative stress promotes TGF-β1-induced epithelial-to-mesenchymal transition in human peritoneal mesothelial cells
Source: Cell Death Dis. 2024 May 28;15(5):365. doi: 10.1038/s41419-024-06753-z (PMC11133371; doi:10.1038/s41419-024-06753-z)

Figure 1. Increased epithelial-to-mesenchymal transition (EMT) via activation of the Smad2/3 signaling pathways and NOX2/4-induced ROS generation in HPMCs treated with TGF-β1. (**A**) TGF-β1 treatment (2 [T2] and 5 [T5] ng/mL) increased mRNA expression of the profibrotic mesenchymal markers (E-cadherin, fibronectin, and α-SMA) in HPMCs. (**B**, **C**) TGF-β1 treatment (2 and 5 ng/mL) increased protein levels of the profibrotic mesenchymal markers and activated the phosphorylation of Smad2/3 signaling in HPMCs. (**D**) TGF-β1 treatment (2 and 5 ng/mL) increased the mRNA expression of *NOX2/4* and *P22phox* in HPMCs after 48 h. (**E**) TGF-β1 induced ROS generation, which was measured using DCF-DA 1 h after TGF-β1 treatment (2 and 5 ng/mL). (**F**) TGF-β1 induced H_2O_2 generation 24 h after treatment (2 and 5 ng/mL). The data are presented as mean ± standard error. n = 4 per group. $^{*}P < 0.05$ vs. control (C); $^{**}P < 0.01$ vs. control; and $^{***}P < 0.001$ vs. control.

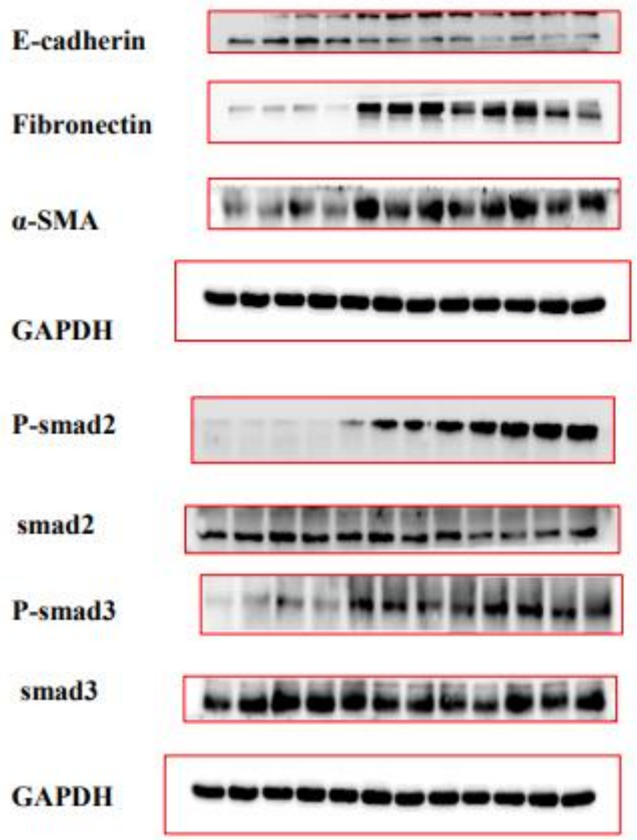

Figure 2. TGF-β1 induced autophagy activation in HPMCs, which was confirmed using transmission electron microscopy (TEM), an autophagy flux assay, and western blotting analysis. (**A**) Representative TEM images of the autophagic morphology. Autophagosomes were frequently observed after TGF-β1 treatment (2 and 5 ng/mL) in HPMCs. The red boxed portion is shown at high magnification on the right. The red arrow indicates the autophagosomes. (**B**) TGF-β1-induced autophagy was evaluated by staining using a Cyto-ID Autophagy Detection Kit. Rapamycin (0.5 μM; Rap) was used as a positive control. The stained cells were observed and photographed under fluorescence microscopy (blue, nucleus/ Hoechst 33342; green, autophagosomes/ Cyto-ID). (**C**) The intensity of the Cyto-ID green was quantified using a plate reader. (**D**) TGF-β1 (2 and 5 ng/mL) increased the mRNA expression of *Beclin 1*, *LC3B*, and *ATG5*, and decreased the mRNA expression of p62. (**E**, **F**) TGF-β1 (2 and 5 ng/mL) increased the protein levels of Beclin 1, LC3B, and ATG5, and decreased the protein levels of p62. The results were calculated as values relative to the control. Data are presented as mean ± standard error (SE). n = 4 per group. $^{*}P < 0.05$ vs. control; $^{**}P < 0.01$ vs. control; and $^{***}P < 0.001$ vs. control.

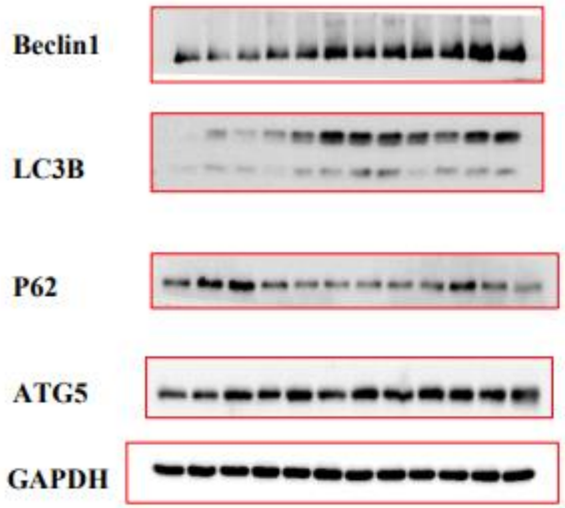

Figure 3. The effects of 3-methyladenine (3-MA) on TGF-β1-induced autophagy activation and epithelial-to-mesenchymal transition (EMT) in HPMCs. (**A**) TGF-β1 (2 and 5 ng/mL) induced autophagy activation but co-treatment with 3-MA (2 mM; 3-MA+T2 and 3-MA+T5) decreased autophagy activation. This was confirmed using a Cyto-ID Autophagy Detection Kit. (**B**, **C**) 3-MA treatment (2 mM) suppressed TGF-β1 (2 and 5 ng/mL) and induced autophagy activation. This was confirmed by western blotting analysis, which revealed decreases in Beclin 1, LC3B, and ATG5, and an increase in p62 levels. Protein levels of the mesenchymal markers fibronectin and α-SMA were decreased by 3-MA in TGF-β1-treated HPMCs. (**D**) Representative immunofluorescence images showing LC3 staining of TGF-β1-induced autophagy activation and EMT in HPMCs. (**D, upper phase**) Representative immunofluorescence images showing LC3B staining of TGF-β1-induced autophagy activation. Positive control cells were treated with 30 μM chloroquine for 16 h. Arrows indicate autophagic flux. (**D, lower phase**) Representative immunofluorescence images showing α-SMA staining of TGF-β1-induced EMT. Arrows indicate the lamellipodia. Scale bar = 40 μm.
The data are presented as mean ± standard error (SE). n = 4 per group. $^{*}P$ <0.05 vs. control; $^{**}P$ < 0.01 vs. control; $^{***}P$ < 0.001 vs. control; $^{\#}P$ < 0.05 vs. TGF-β1 2 ng/mL; $^{\#\#}P$ < 0.01 vs. TGF-β1 2 ng/mL; $^{\#\#\#}P$ < 0.001 vs. TGF-β1 2 ng/mL; ^{+}P < 0.05 vs. TGF-β1 5 ng/mL; ^{++}P < 0.01 vs. TGF-β1 5 ng/mL; and ^{+++}P < 0.001 vs. TGF-β1 5 ng/mL.

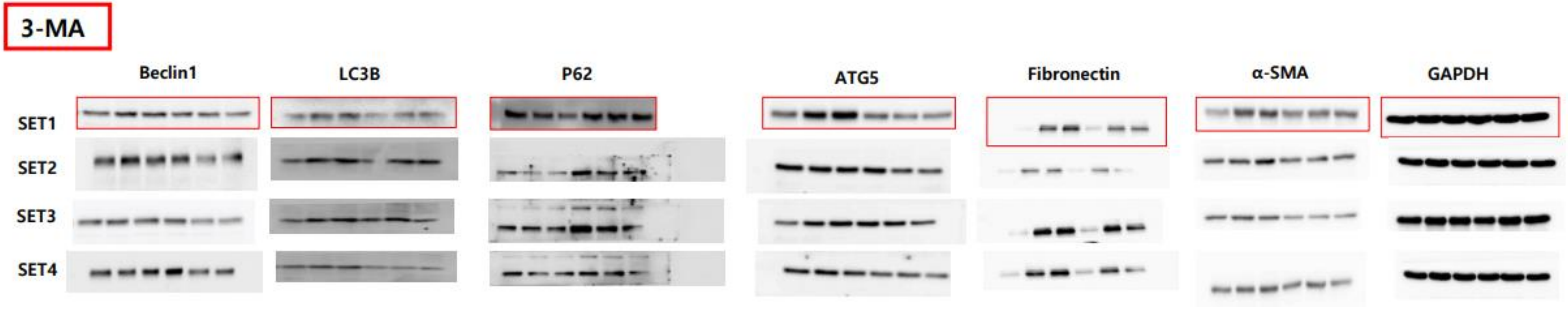

Figure 4. The effects of NOX4 inhibition on TGF-β1-induced autophagy activation and epithelial-to-mesenchymal transition (EMT) in HPMCs. (**A**) The expression of NOX4 and autophagy marker protein levels following GKT137831 (20 μM; GKT20) and TGF-β1cotreatment. (**B**) GKT137831 (20 μM) co-treatment resulted in lower NOX4 protein levels than TGF-β1 (5 ng/mL) treatment alone. Cotreatment with GKT137831 (20 μM) suppressed TGF-β1-induced autophagy activation. This was confirmed by western blotting analysis, which revealed decreased Beclin 1, LC3B, and ATG5, and increased p62 levels. The protein levels of the mesenchymal markers fibronectin and α-SMA were decreased by GKT137831 in TGF-β1-treated HPMCs. **(C)** TGF-β1 (2 and 5 ng/mL) induced autophagy activation and co-treatment with GKT137831 (20 μM; GKT20+T2 and GKT20+T5) decreased autophagy activation. This was confirmed using a Cyto-ID Autophagy Detection Kit. **(D)** Representative immunofluorescence images showing LC3B staining of TGF-β1-induced autophagy activation (**upper phase**). Positive control cells were treated with 30 μM chloroquine for 16 h. The arrows indicate autophagic flux. Representative immunofluorescence images showing α-SMA staining of TGF-β1-induced EMT (**lower phase**). The arrows indicate the lamellipodia. Scale bar = 40 μm.
Data are presented as mean ± standard error (SE). n = 4 per group. $^{*}P$ <0.05 vs. control; $^{**}P$ < 0.01 vs. control; $^{***}P$ < 0.001 vs. control; $^{\#}P$ < 0.05 vs. TGF-β1 2 ng/mL; $^{\#\#}P$ < 0.01 vs. TGF-β1 2 ng/mL; ^{+}P < 0.05 vs. TGF-β1 5 ng/mL; ^{++}P < 0.01 vs. TGF-β1 5 ng/mL; and ^{+++}P < 0.001 vs. TGF-β1 5 ng/mL.

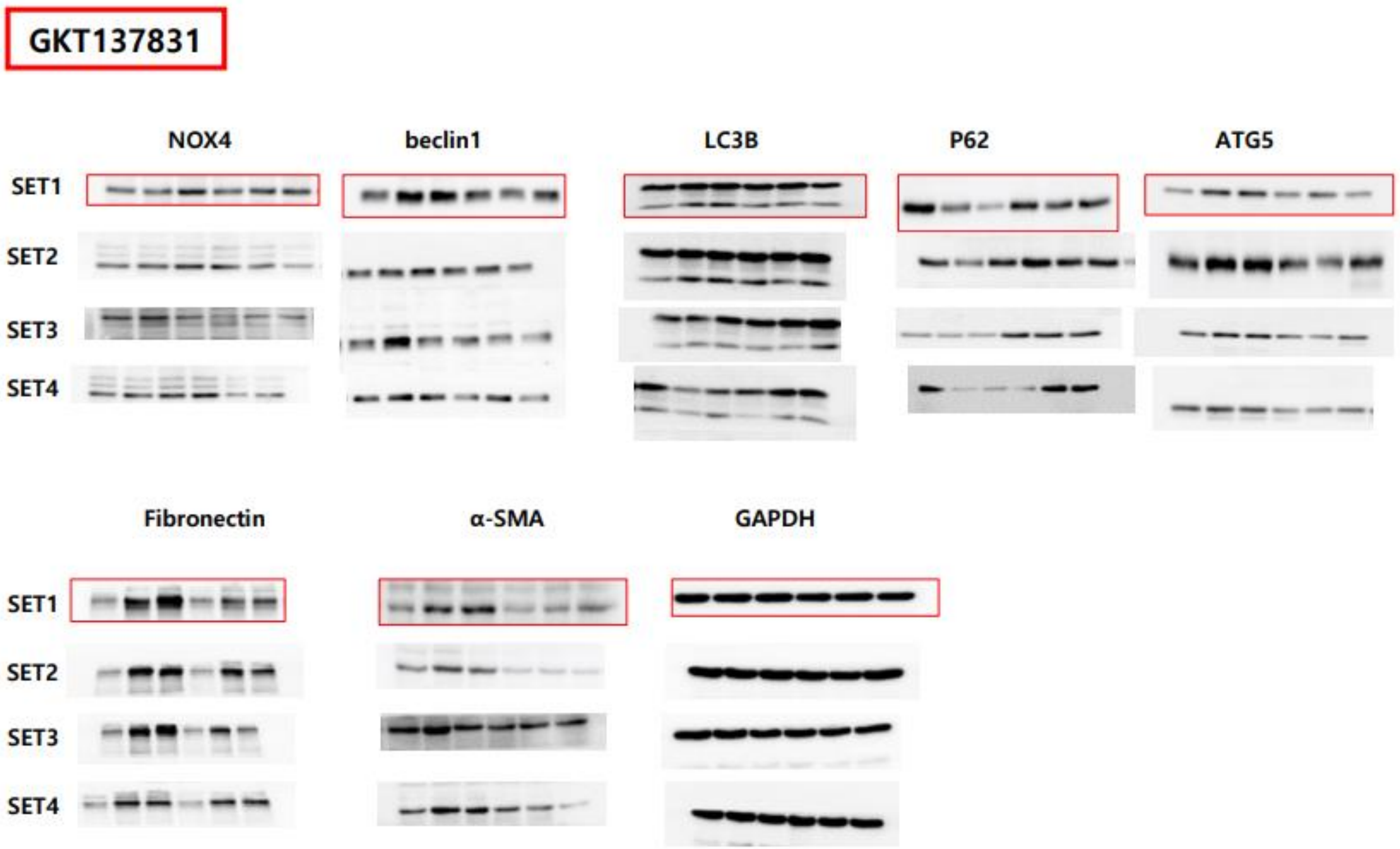

Figure 6. Autophagy inhibition by 3-MA treatment inactivated the Smad2/3, PI3K/Akt, and ERK pathways in HPMCs. 3-MA treatment (2 mM) decreased the TGF-β1 (2 and 5 ng/mL)-induced phosphorylation of Smad2/3 signaling for EMT (**A**, **B**), PI3K and Akt signaling for autophagy (**A**, **C**), and the ERK signaling of the MAPK pathway (**A**, **D**).
The data are presented as mean ± standard error (SE). n = 4 per group. $^{*}P$ <0.05 vs. control; $^{**}P$ < 0.01 vs. control; $^{***}P$ < 0.001 vs. control; $^{\#}P$ < 0.05 vs. TGF-β1 2 ng/mL; $^{\#\#}P$ < 0.01 vs. TGF-β1 2 ng/mL; ^{+}P < 0.05 vs. TGF-β1 5 ng/mL; ^{++}P < 0.01 vs. TGF-β1 5 ng/mL; and ^{+++}P < 0.001 vs. TGF-β1 5 ng/mL.

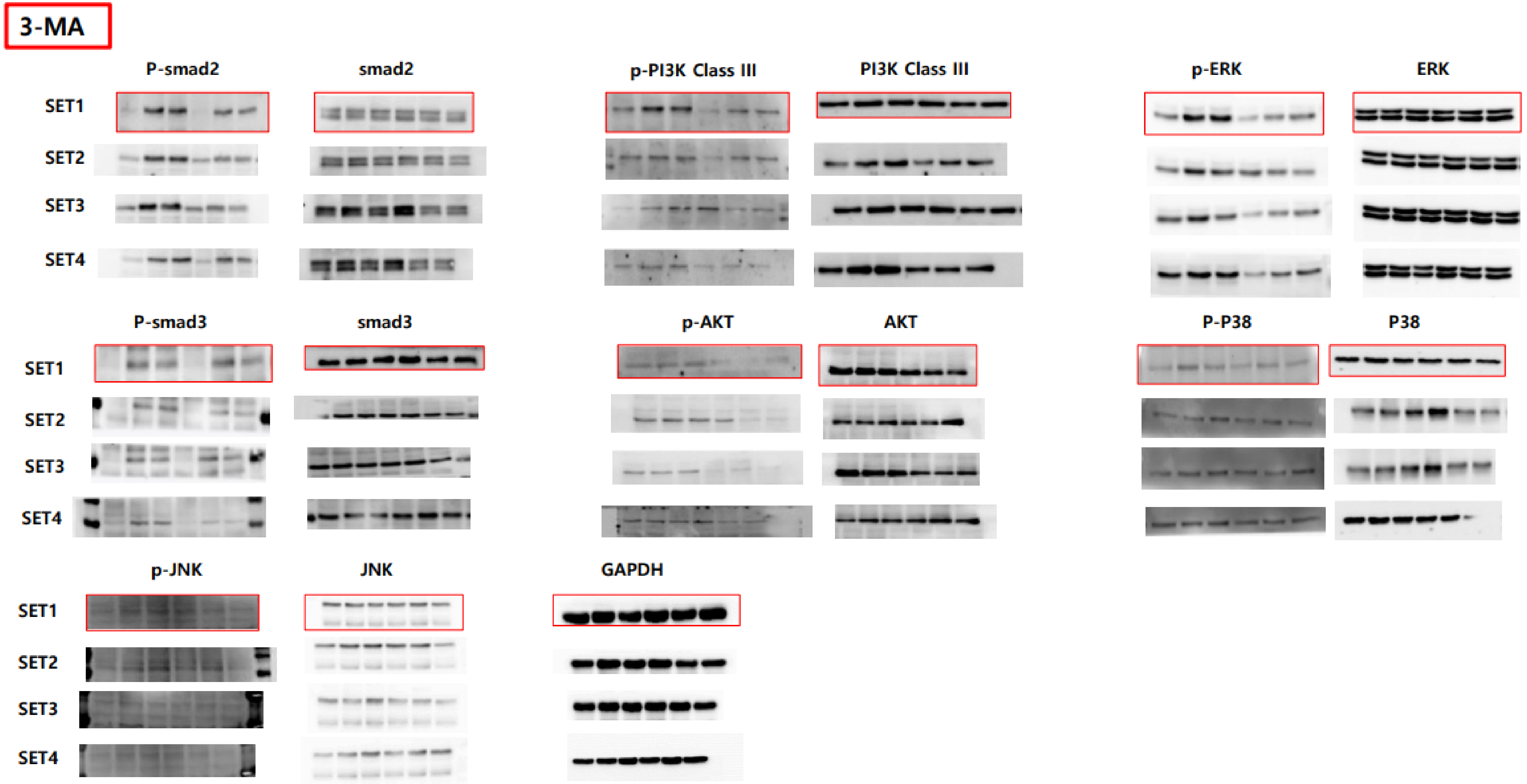

Figure 7. NOX4 inhibition with GKT137831 inactivated the Smad2/3, PI3K/Akt, and P38 pathways in HPMCs. GKT137831 (20 μM) decreased the TGF-β1 (2 and 5 ng/mL)-induced phosphorylation of Smad2/3 signaling for EMT (**A**, **B**), PI3K and Akt signaling for autophagy (**A**, **C**), and the P38 signaling of the MAPK pathway (**A**, **D**).
The data are presented as mean ± standard error (SE). n = 4 per group. $^{*}P < 0.05$ vs. control; $^{**}P < 0.01$ vs. control; $^{***}P < 0.001$ vs. control; $^{\#}P < 0.05$ vs. TGF-β1 2 ng/mL; $^{\#\#}P < 0.01$ vs. TGF-β1 2 ng/mL; $^{\#\#\#}P < 0.001$ vs. TGF-β1 2 ng/mL; $^{+}P < 0.05$ vs. TGF-β1 5 ng/mL; $^{++}P < 0.01$ vs. TGF-β1 5 ng/mL; $^{+++}P < 0.001$ vs. TGF-β1 5 ng/mL.

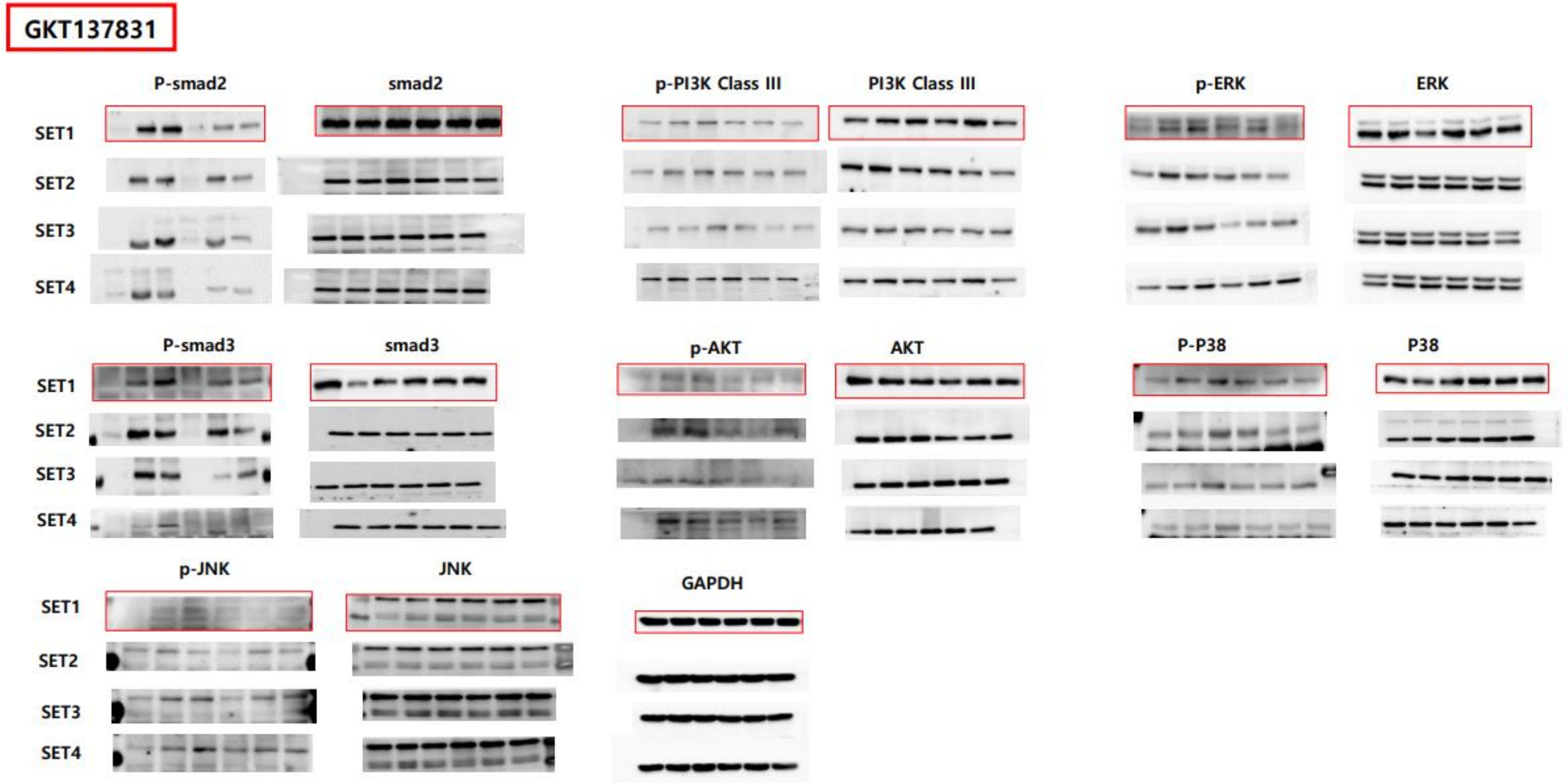

Supplementary Figure S1.

Figure S1. Effect of *ATG5* gene silencing on TGF-β1-induced autophagy activation and EMT in HPMCs. (**A**, **B**) *ATG5* gene silencing suppressed the TGF-β1 (2 [siATG5+T2] and 5 [siATG5+T5] ng/mL)-induced autophagy activation, which was confirmed via Western blotting analysis with a decrease in the ATG5, Beclin 1, and LC3B levels and an increase in the p62 levels. Protein levels of the mesenchymal markers (fibronectin and α-SMA) were decreased by *ATG5* gene silencing in TGF-β1-treated HPMCs. The data are presented as the mean ± standard error (SE); n = 4 per group. $^{*}P < 0.05$ vs. control small interfering RNA (siCon); $^{**}P < 0.01$ vs. siCon; $^{***}P < 0.001$ vs. siCon; $^{\#}P < 0.05$ vs. siCon+TGF-β1 2 ng/mL (siCon+T2); $^{\#\#}P < 0.01$ vs. siCon+T2; $^{\#\#\#}P < 0.001$ vs. siCon+T2; $^{+}P < 0.05$ vs. siCon+TGF-β1 5 ng/mL [siCon+T5]; $^{++}P < 0.01$ vs. siCon+T5; and $^{+++}P < 0.001$ vs. siCon+T5.

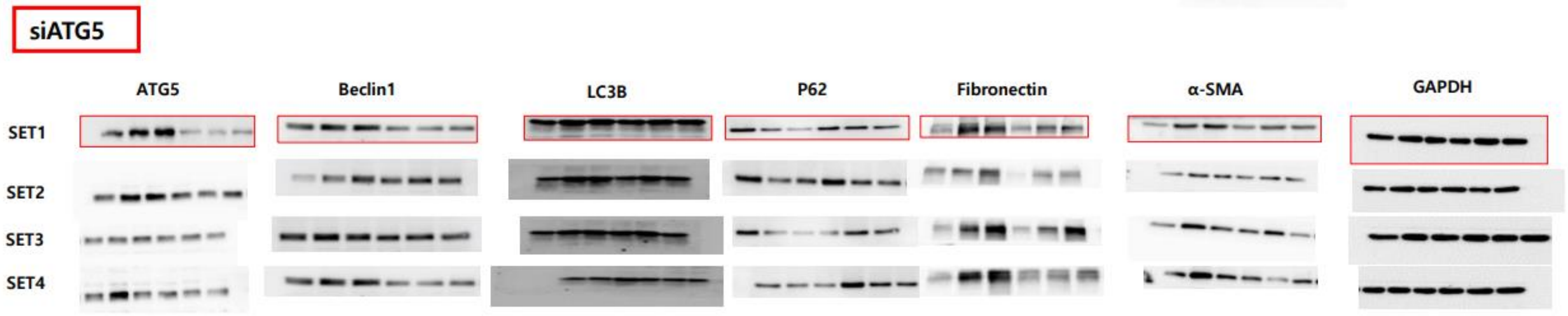

Supplementary Figure S3.

Figure S3. Autophagy inhibition by *ATG5* gene silencing inactivates the Smad2/3, PI3K/AKT, and ERK pathways in HPMCs. *ATG5* gene silencing decreased the TGF-β1 (2 [siATG5+T2] and 5 [siATG5+T5] ng/mL)-induced phosphorylation of Smad2/3 signaling for EMT (**A**, **B**), PI3K and AKT signaling for autophagy (**A**, **C**), and ERK and P38 signaling of the MAPK pathway (**A**, **D**). The data are presented as the mean ± standard error (SE); n = 4 per group. $^{*}P < 0.05$ vs. control small interfering RNA (siCon); $^{**}P < 0.01$ vs. siCon; $^{***}P < 0.001$ vs. siCon; $^{\#}P < 0.05$ vs. siCon+TGF-β1 2 ng/mL (siCon+T2); $^{\#\#}P < 0.01$ vs. siCon+T2; $^{+}P < 0.05$ vs. siCon+TGF-β1 5 ng/mL (siCon+T5); $^{++}P < 0.01$ vs. siCon+T5; and $^{+++}P < 0.001$ vs. siCon+T5.

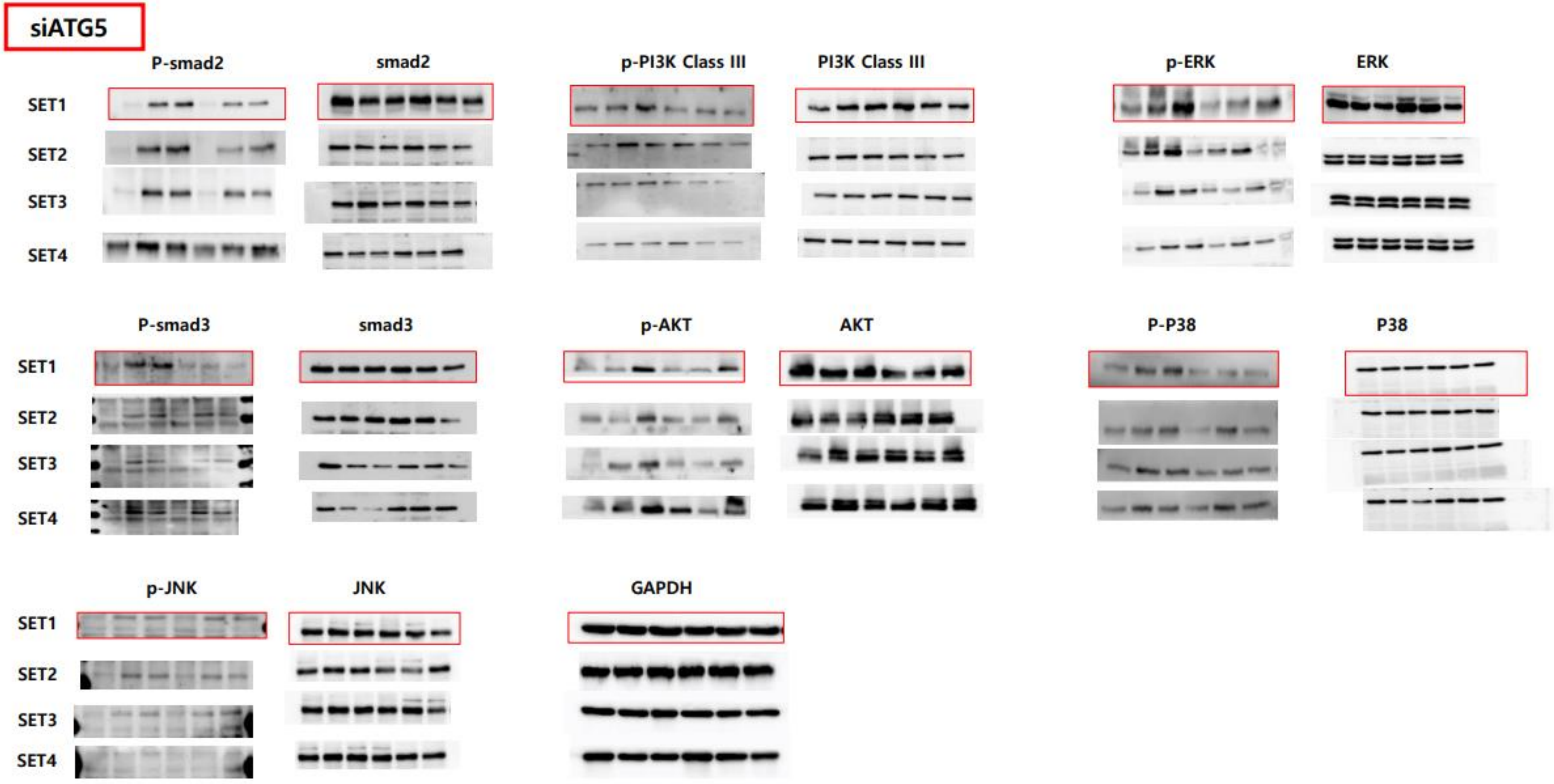

Supplement: Supplementary file 2 — Original Data File [file 41419_2024_6753_MOESM2_ESM.pdf]
